# Two cases of possible transmitted drug-resistant HIV: likely HIV superinfection and unmasking of pre-existing resistance

**DOI:** 10.1177/0956462415571671

**Published:** 2016-01

**Authors:** Fabiola Martin, John Lee, Emma Thomson, Nick Tarrant, Antony Hale, Charles J Lacey

**Affiliations:** 1 Centre for Immunology and Infection, Department of Biology, University of York, York, UK; 2 Genitourinary Department, Josephine Butler Centre King Street Health Centre, Wakefield, UK; 3 Centre for Virus Research, University of Glasgow, Glasgow, UK; 4 Genitourinary Department, York Teaching Hospital, York, UK; 5 Department of Virology, Leeds Teaching Hospitals Trust, Leeds, UK

**Keywords:** HIV, AIDS, Transmitted drug resistance, superinfection, dual infection, minority species, syphilis (*Treponema pallidum*), antiviral, highly active antiretroviral therapy, high-risk behaviour, hepatitis C, viral disease

## Abstract

In the UK, patients undergo HIV viral load and genotype testing before they are prescribed antiretroviral therapy. The genotype test guides clinicians in prescribing antiretroviral therapy with maximum efficacy against the patient’s specific viral strain. HIV viral load escape under antiretroviral drug therapy, to which the virus was thought to be genotypically susceptible, is commonly observed in patients with poor adherence. We observed early viral escapes in two-newly diagnosed patients, during antiretroviral treatment, with different sequences compared to their original viral resistance test and who reported excellent adherence to and tolerance of their therapy. HIV superinfection with a new viral strain was identified in a patient with multiple risk factors and co-infections with sexually transmitted infections. The second patient was a case of the emergence of primary resistant virus under drug pressure. Both suppressed their virus promptly after treatment switch.

## Case 1

**Figure 1.**
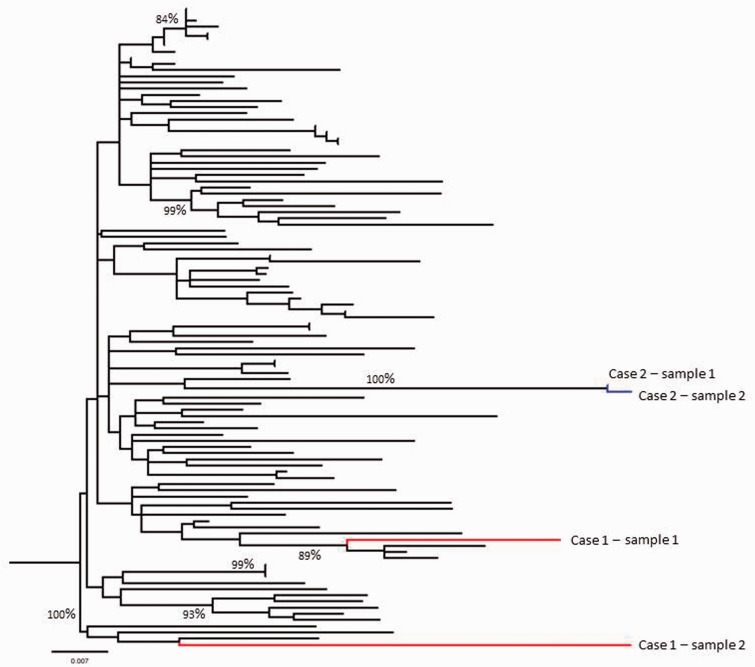
Molecular phylogenetic analysis by maximum likelihood method. Branches derived from Case 1 are shown in red while those from Case 2 are shown in blue. Branches in black were derived from reference sequences obtained from the NCBI database. The evolutionary history was inferred by using the maximum likelihood method based on the General Time Reversible model.^14^ The tree with the highest log likelihood (−7594. 0650) is shown. The percentage of trees in which the associated taxa clustered together is shown next to the branches. Initial tree(s) for the heuristic search were obtained automatically by applying Neighbor-Join and BioNJ algorithms to a matrix of pair-wise distances estimated using the maximum composite likelihood approach and then selecting the topology with superior log likelihood value. A discrete Gamma distribution was used to model evolutionary rate differences among sites (five categories [+*G*, parameter = 0.2316]). The rate variation model allowed for some sites to be evolutionarily invariable ([+*I*], 51.4057% sites). The tree is drawn to scale, with branch lengths measured in the number of substitutions per site. The analysis involved 104 nucleotide sequences. Codon positions included were 1st+2nd+3rd+Non-coding. All positions with less than 95% site coverage were eliminated. That is, fewer than 5% alignment gaps, missing data and ambiguous bases were allowed at any position. There were a total of 1006 positions in the final dataset. Evolutionary analyses were conducted in MEGA6.^15^ Bootstrap values >70% are illustrated on the tree (1000 bootstrap replicates). Final editing was carried out using Figtree v1.4.2.

A 41-year-old, White British, man who has sex with men (MSM) presented complaining of drenching night sweats and fatigue of recent onset. He reported unprotected oral and anal intercourse with casual partners and had recently been diagnosed with hepatitis C virus (HCV). He gave no travel history of note. He was diagnosed with and treated for early, secondary syphilis. His HCV genotype was 1a (HCV RNA: 3.87 × 104 copies/mL) and his HIV serology testing was negative. Four weeks later, he presented with night sweats, anorexia and dysphagia. Oesophageal candidiasis was suspected and treated with fluconazole 50 mg once daily for seven days. HIV-1 antibodies were positive (HIV viral load [VL]: 10 million copies/mL, Log^10^ >5.8, CD4 count: 263 × 10^6^/L, 21%). Acute HIV sero-conversion syndrome was diagnosed. Sequencing of protease (PR) and first 234 amino acids of the reverse transcriptase (RT) genes indicated a fully antiretroviral-susceptible HIV-1 subtype B strain with minor PR and RT differences to the HIV reference strain HXB2 (K20R, E35D, N37E, L63P; V8I, V90I, I142T, S162D, I178M). HCV RNA was undetectable, suggesting spontaneous clearance of the HCV. Sixteen weeks later, despite initial recovery, the CD4 had declined to 185 × 10^6^/L (12%). The HIV-1 VL was 167,200 copies/mL (Log^10^ 5.2). Abacavir, lamivudine and nevirapine were commenced and the VL suppressed to 270 copies /mL (Log^10^ 2.4) at 12 weeks. Four weeks later, the patient presented feeling unwell with night sweats. On examination, he had right-sided inguinal lymphadenopathy and enlarged liver and a palpable spleen. A pale macular rash was visible on the trunk and extremities. He was diagnosed with rectal *Neisseria gonorrhoeae* and urethral *Chlamydia trachomatis*, his HCV RNA remained negative. However, the HIV VL had risen to 14,900 copies/ml (Log10 4.2) despite good reported adherence and tolerance. HIV genotyping revealed HIV-1 subtype B with multiple new mutations: PR: K14R, R83K, RT: A98S, K101E, M184I, G190A, T215D, I135T, D177E, I178L, V179I, T200A, I202V, Q207K, R211K. This virus, showed high-level resistance to lamivudine and nevirapine and low-level resistance to abacavir. The sequences were highly divergent exhibiting 45 nucleotide differences of which 20 were non-synonymous including the four resistance-associated mutations (Figure 1). Considering the history of repeated unprotected sex and co-infections with sexually transmitted infections together with the number of changes in non-resistance mutations HIV superinfection was suspected. This was confirmed by phylogenetic analysis. Highly active antiretroviral therapy (HAART) was changed to abacavir, lamivudine, zidovudine and boosted atazanavir. The HIV VL was <50 copies/mL (log^10^ 1.6) within three weeks. Fifteen months later, routine tests revealed newly raised AST (73IU/L) and bilirubin (89 µmol/L) levels and HCV RNA levels of 2.6 × 10^6^ copies/mL. The two HCV strains were identical in the 135 base region of the 5’ NCR. Considering the previous HCV RNA negative tests and ongoing unprotected sex, HCV reinfection was thought to be more likely than reactivation.

## Case 2

A 41-year-old, White British MSM receiving treatment for *Pneumocystis jiroveci* pneumonia was diagnosed with HIV. The virus was subtype B and fully antiretroviral susceptible (VL 260,000 copies/mL, log^10^ 5.4, CD4 count 4 × 10^6^/L, 2.5%). He was commenced on tenofovir, emtricitabine and efavirenz. He improved clinically, but the CD4 count plateaued at 11 × 10^6^/L, 2.6%) and the VL only decreased to 88,000 copies/mL (log^10^ 4.9) at 4 weeks and stalled at 56,000 copies/mL (log^10^ 4.7) at 12 weeks. He reported no side-effects, no new sexual contacts and 100% adherence to HAART. He gave no travel history of note. Resistance testing revealed K65KR, M184V, K101E, Y181CY and G190CS. Comparison of the two sequences revealed only four out of 1007 sequences nucleotide changes between baseline and the second test, excluding residues exhibiting mixed bases, of which three resulted in amino acid changes within known resistance foci at RT residues 101, 184 and 190. The fourth was a synonymous nucleotide change. Based on the fact that the patient denied lack of exposure, reported good adherence and the commonly under drug pressure observed mutations, this lack of sufficient VL response was thought to be due to an unmasking of a primary minority species virus under drug pressure, rather than HIV superinfection. The patient's treatment was switched to zidovudine, lamivudine, raltegravir and boosted darunavir. Twenty-four weeks later he was feeling well and his VL was 41 copies/mL (log^10^ 1.6) and his CD4 count 155 × 10^6^/L, 17%.

## Discussion

We report viral escape as a consequence of potential transmitted drug resistance due to HIV-1 superinfection and the unmasking of a primary minority species resistant virus under drug pressure. In both case, viral drug resistance was discovered following an insufficient immunological and virological response. Both patients had received information on transmission and prevention of sexually transmitted infections as well as HIV superinfection and adherence to and side-effects of antiretroviral medication. We routinely provide free condoms and advise to refrain from sex during the sero-conversion period.

Poor adherence, ineffective HAART, HIV superinfection and unmasking of primary resistant mutants are known to be associated with lack of sufficient virological response.^1^ The latter two are rare but should be considered in patients with good adherence to HAART. A detailed history and HIV VL measurement, genotype and phylogenetic analysis, especially ultradeep pyrosequencing,^2^ are useful in distinguishing the cause of viral escape. HIV superinfection with a different virus can be acquired after primary seroconversion.^3,4^ It is associated with early HIV infection^5,6^ and estimated to occur at a rate of around 5 per 100 person-years.^2^ It may lead to the acquisition of drug resistance,^7^ viral escape, may necessitate treatment switch and be associated with disease progression.^8^ In our case, repeat genotyping detected the distinctly different new virus and guided a successful treatment switch. We believe that the archived resistance in case 2 was 194V and 101E as these were not present in mixtures and that 65R and 181C then rapidly emerged as indicated by these being mixed with wild type. The residue at 190 was also mixed but not with wild type, and we cannot be sure whether one or both of these were archived or emerged in different quasi-species. We were not able to conduct ultradeep sequencing, since it is not yet validated at our routine virology service. However, the high divergence in the first case and the lack of divergence in the second case make superinfection in the first case and the unmasking of a previous resistant variant in the second case, the most likely explanation (Figure 1).

Random samples of treatment-naïve HIV-infected subjects harboured 5%, 3% and 3% primary resistant HIV strains to NNRTIs, NRTIs and PIs, respectively.^9^ In the absence of drug pressure, the mutated virus may revert back to wild-type and not be detectable with simple genotyping.^10,11^ The overall rate of loss of mutations was reported to be 18 per 10 person-years follow-up, with large variation in the loss of NRTI mutations (M184V rapid, M41L & thymidine analogue mutations [TAMs] stable) over time and a more rapid loss of NNRTI and PI mutations compared to TAMs.^12^ Using these data, in our second case, the M184V and Y181C may have been lost quicker than the remaining three; however, they re-emerged under drug pressure.

We support the current practice:^13^ advising patients to avoid unprotected sex, measuring VL at baseline at 4, 8, 12 and 24 weeks, tailoring treatment to the viral genotype, repeating genotype in viral escape and switching treatment in response to the new genotype.

## Consent

Both patients have consented for the cases to be published.
